# The Na^+^/H^+^ exchanger in metastasis

**DOI:** 10.18632/aging.101002

**Published:** 2016-07-19

**Authors:** Schammim Ray Amith, Larry Fliegel

**Affiliations:** Department of Biochemistry, University of Alberta, Edmonton, Alberta, T6G 2H7, Canada

**Keywords:** Na+/H+ exchanger, metastasis, triple-negative breast cancer

Triple-negative breast cancer, in which cells lack the expression of estrogen, progesterone, and human epidermal growth factor 2 receptors, is a clinical subtype of breast cancer commonly associated with poor prognosis. It tends to be aggressively metastatic with a high recurrence rate and little response to standard chemotherapy [1]. Mounting evidence suggests that one triggering event in oncogenic transformation is an imbalance in acid-base homeostasis, with an increase in intracellular pH and acidification of the extracellular tumor micro-environment [2]. In breast cancer cells, intra-cellular pH is largely controlled by the action of the Na+/H+ exchanger isoform one (NHE1). Previous reports show that excessive activity of the Na+/H+ exchanger triggers extracellular matrix proteolysis and promotes metastatic invasion by breast cancer cells [3]. We recently reported that this trigger of metastasis is critical in triple-negative breast cancer cells, but not as crucial in other types of breast cancer [4]. We generated an NHE1-knockout of MDA-MB-231 (triple-negative) breast cancer cells. The NHE1-knockout cells proliferated normally but had greatly reduced rates of migration and invasion in in vitro assays. Growth of the knockout cells in xenograft tumor assays was also decreased dramatically compared to cells with the Na+/H+ exchanger. Specific inhibition of exchanger activity in cells expressing NHE1 resulted in the increased efficacy of paclitaxel in promoting cell death and limiting rates of migration and invasion in different triple-negative breast cancer cells. However, this effect was not seen in non-triple-negative breast cancer cells. As a follow up, we directly examined the role of regulatory mechanisms responsible for the activation of NHE1 in metastasis [5]. Site-specific mutagenesis made an activated from of the Na+/H+ exchanger and this change promoted cell migration, invasion, and spheroid growth. Mutation of the p90RSK phosphorylation site on NHE1's cytosolic C-terminal domain to a non-phosphorylatable amino acid altered the morphology of mesenchymal MDA-MB-231 cells to a less invasive, epithelial-like phenotype compared to cells expressing wild-type NHE1. Notably, pharma-cological inhibition of p90RSK also inhibited the metastatic potential of invasive MDA-MB-231 cells, but not of non-invasive Hs578T triple-negative breast cancer cells.

Overall, our data suggest that the Na+/H+ exchanger plays a key role in the metastasis of breast cancer cells. However, its function appears to be more critical in the invasive triple-negative breast cancer subtype. We propose that it is the excessive activity of NHE1 during carcinogenesis that is essential in invasion and metastasis, and in promoting a change to a more invasive cell phenotype. While our data were supported by the examination of several triple-negative cell models, it is not yet known whether this occurs in most or all triple-negative cell types or if this observation can be exploited in therapy. Additionally, while we demonstrated that p90RSK inhibition has beneficial effects in cells in culture, it is not clear if this inhibition of the Na+/H+ exchanger is a clinically viable prospect. Furthermore, NHE1 has multiple regulatory sites for kinase phosphorylation and interactions with intracellular protein and lipid binding partners. Many of these sites of regulation are not well defined in triple-negative breast cancer cells, so that the call for further investigation remains.
